# Evaluating the recovery of pan-susceptible and antibiotic-resistant *Escherichia coli* in synthetic test agricultural water using membrane filtration and colilert methods

**DOI:** 10.1186/s13104-025-07423-9

**Published:** 2025-08-20

**Authors:** Zirui Ray Xiong, Ajani A. Brooks, Ellen Gabriel, Alan Gutierrez, Shayla B. Johnson, Cheryl East, Lisa M. Durso, Elica Moss, Manan Sharma

**Affiliations:** 1 United States Department of Agriculture, Agricultural Research Service, Beltsville Agricultural Research Center, Environmental Microbial and Food Safety Laboratory, Beltsville, MD United States of America; 2 Department of Animal and Food Sciences, University of Delaware, Newark, DE United States of America; 3 Department of Plant and Soil Science, Alabama A&M University, Huntsville, AL United States of America; 4 United States Department of Agriculture, Agricultural Research Service, Agroecosystems Management Research, Lincoln, NE United States of America

**Keywords:** Agricultural water, Membrane filtration, Colilert assay, Antimicrobial resistance, Escherichia coli, Test methods

## Abstract

Membrane filtration and Colilert assays are commonly used to quantify *Escherichia coli* levels in agricultural water. These methods have not been evaluated in test agricultural water (TAW), a formulation used for assessing chemical sanitizer effectiveness. Quantitative recovery of *E. coli* in TAW may be affected by turbidity levels. TAW was formulated with a pH value of 6.5 at two turbidity levels (0 and 50 NTU). Pan-susceptible and cefotaxime-resistant *E. coli* strains were added separately to TAW (200 CFU/100 mL). Inoculated TAW was either (1) filtered using a 0.45 μm membrane filter (MF) and placed onto Tryptone Bile X-Glucuronide (TBX) agar or CHROMagar ECC (ECC), or (2) tested by IDEXX Quanti-Tray/2000 Colilert. Recovery percentages of *E. coli* at both turbidity levels were similar on TBX and ECC, but pan-susceptible *E. coli* ARS C101 in TAW at 50 NTU on TBX was not quantifiable. For Colilert assays, the recovery percentages of *E. coli* from 0 NTU and 50 NTU TAW were between 93.4 ± 4.4% and 127.0 ± 10.5%. Overall, ECC and Colilert provided quantifiable results for recovery of pan-susceptible and cefotaxime-resistant *E. coli* from artificial agricultural water at different turbidity levels. Higher levels of turbidity affected the ability to quantify *E. coli* by MF with TBX and recovery by Colilert.

**Supplementary Information:**

The online version contains supplementary material available at 10.1186/s13104-025-07423-9.

## Introduction

*Escherichia coli* is commonly found in the intestinal tract of warm-blooded animals, such as cattle, sheep, and swine [1], and its presence commonly signifies fecal pollution in water. *E. coli* levels frequently determine the use and treatment of agricultural water. Agricultural water includes water used for irrigation, application of fertilizers and pesticides, cooling, washing tools, and frost protection [2]. Depending on the source and distribution methods, agricultural water could potentially transfer foodborne pathogens from water to fruits and vegetables grown in the fields. Surface water that is open to the environment is more likely to be contaminated with fecal material and carry human bacterial pathogens, such as *E. coli* O157:H7, *Salmonella* spp., and *Listeria monocytogenes*, posing a risk to fresh fruits and vegetables that are consumed raw or minimally processed.

Microbial testing can provide useful information on the quality of agricultural water. Quantitative levels of *E. coli* in agricultural water affect the decision-making process on whether or not to treat agricultural water with a chemical sanitizer to improve its microbial quality. Even though the presence of generic *E. coli* does not directly correlate with the presence of enteric pathogens, it suggests fecal contamination and the potential presence of pathogens [3, 4]. In recreational water, there are standards established for these fecal indicators based on epidemiological data connecting *E. coli* levels with risks of human gastrointestinal diseases [5]. The criteria recommended by U.S. Environmental Protection Agency (EPA) for *E. coli* MPN levels in recreational water is a geometric mean of 126 CFU per 100 mL and a statistical threshold value of 235 CFU per 100 mL [6].

Additionally, *E. coli* is considered a major reservoir of antimicrobial resistance (AMR) genes [7–9]. While some strains of *E. coli* are pan-susceptible, meaning that they are susceptible to all or most common antibiotics, others are multidrug resistant (MDR), resistant to multiple classes of antibiotics. As a naturally competent organism that can incorporate external DNA in the environment, *E. coli* is able to donate and receive AMR genes to or from other bacteria through horizontal gene transfer [10–12]. Monitoring antimicrobial resistance, prevalence, and distribution of *E. coli* in the environment, specifically in water, contributes to our understanding of AMR dissemination in the environment and the One Health approach for AMR surveillance.

There are two commonly used types of culture-based methods to quantify *E. coli* in agricultural water: Membrane filtration (MF) and enzyme substrate test combined with Most Probable Number (MPN) method. MF is based on filtering a fixed volume of water through membrane filter and placing it onto selective agar. After filtration, bacteria in water are retained on the filter due to size exclusion. By incubating the filter on selective and differential media, such as Tryptone Bile X-glucuronide (TBX) or CHROMagar ECC (ECC), presumptive *E. coli* colonies are identified by their appearance and enumerated. TBX agar identifies *E. coli* based on its production of β-D-glucuronidase, an enzyme highly specific for *E. coli* [13]. The IDEXX Quanti-Tray/2000 Colilert assay uses defined substrate technology and simultaneously detects both total coliforms and *E. coli* based on the enzymatic activity of β-D-galactosidase and β-D-glucuronidase [14], respectively. β-D-glucuronidase activity is identified by fluorescence (emission at 365 nm). The Colilert assay has been used to determine *E. coli* MPN levels in recreational water, wastewater, and drinking water [15]. It is considered a scientifically valid testing methodology for quantifying generic *E. coli* in agricultural water according to the FDA Produce Safety Rule (21 CFR 112). Several previous studies have modified the Colilert assay to target the recovery of antibiotic-resistant *E. coli* in wastewater and surface water samples [15, 16]. Cefotaxime is commonly added to media to recover extended-spectrum beta-lactamase (ESBL)-producing *E. coli* from water samples. ESBL-producing *E. coli* strains are resistant to commonly used antibiotics, such as penicillin, oxyimino-cephalosporins (cefotaxime, ceftazidime, ceftriaxone, cefuroxime, cefixime), and monobactams (aztreonam) and are considered a global health threat [17–20]. Detecting and monitoring the prevalence of ESBL-producing *E. coli* in the environment can assess the risk of municipal and agricultural discharges disseminating AMR in the environment in an effort to mitigate antimicrobial resistance dissemination [15, 16].

Test agricultural water (TAW) is a formulation proposed by the U.S. FDA and EPA for assessing the effectiveness of chemical sanitizer treatments on bacterial pathogens in agricultural water [21]. This formula contains standard test dust, to simulate turbidity of natural water; humic acid, to add total organic carbon (TOC); and commercial sea salt to add total dissolved solids (TDS). Considering the highly variable chemical properties and microbiological background of natural agricultural water, the TAW formulation can be a standardized matrix to simulate agricultural water. To our knowledge, no previous studies have compared the recovery of pan-susceptible and antibiotic-resistant *E. coli* using different enumeration methods in a standardized matrix for agricultural water. To assess the method validity to recover pan-susceptible and ESBL-producing *E. coli*, MF with selective media TBX and ECC were compared to IDEXX Quanti-Tray/2000 Colilert assay in TAW with and without the addition of test dust.

## Materials and methods

Pan-susceptible *E. coli* ARS C101 and cefotaxime- and tetracycline-resistant *E. coli* ARS C301 were originally isolated from cattle [22]. Strains C101 and C301 were inoculated in 10 mL tryptic soy broth (TSB) and TSB with 4 µg/mL cefotaxime (TSBC), respectively, and incubated overnight at 37 °C. Overnight cultures of C101 and C301 was diluted to ca. 100 cells per 100 µL based on absorption readings at 600 nm wavelength with a spectrophotometer (Thermo Scientific Genesys 20) and plate count on tryptic soy agar (TSA).

Synthetic test agricultural water (TAW) was prepared using a modified EPA protocol [21]. PTI Arizona test dust (Nominal 0–70 micron, PTI Powder Technology) was used to achieve a turbidity level of 50 NTU, while all other components were used according to Table S1 (supplementary information). TAW was mixed before pH and turbidity measurements were taken to prevent dust settling. The pH value was measured using an Orion 3 Star benchtop pH meter (Thermo Scientific). The turbidity of the TAW was measured using the Orion AQ4500 Turbidity Meter (Thermo Scientific).

Sterile TAW (100 mL) at pH 6.5 with either no added turbidity (0 NTU) or 50 NTU was inoculated with 200 µL (ca. 200 cells) of either *E. coli* ARS C101 or C301 diluted overnight culture. Inoculated TAW was filtered through a 0.45 μm cellulose ester membrane (Fisher Scientific), and the filter was placed onto either TBX or ECC. Cefotaxime-resistant *E. coli* (ARS 301)was recovered on TBX or ECC containing 4 µg/mL cefotaxime. Plates were incubated at 37 °C for 24 h and the colonies on the surface of membrane filter were enumerated. *E. coli* levels in inoculated TAW were quantified with IDEXX Quanti-Tray/2000 Colilert Assay according to the manufacturer’s instruction. To recover *E. coli* ARS C301, a final concentration of 4 µg/mL cefotaxime was added to 100 mL TAW sample. After incubation at 35 °C for 24 h, positive wells in the Quanti-Tray were determined by observing fluorescence at a wavelength of 365 nm using a hand-held ultraviolet light, and the MPN value of *E. coli* in 100 mL TAW was determined. All experiments were performed in triplicate. Data visualization and analysis was performed in R version 4.2.2 (www.r-project.org), RStudio version 2025.05.1–513, and packages *tidyverse* version 2.0.0, *dplyr* version 1.1.4, *stats* version 4.4.1, *rstatix* version 0.7.2.

## Results and discussion

**Fig. 1:**
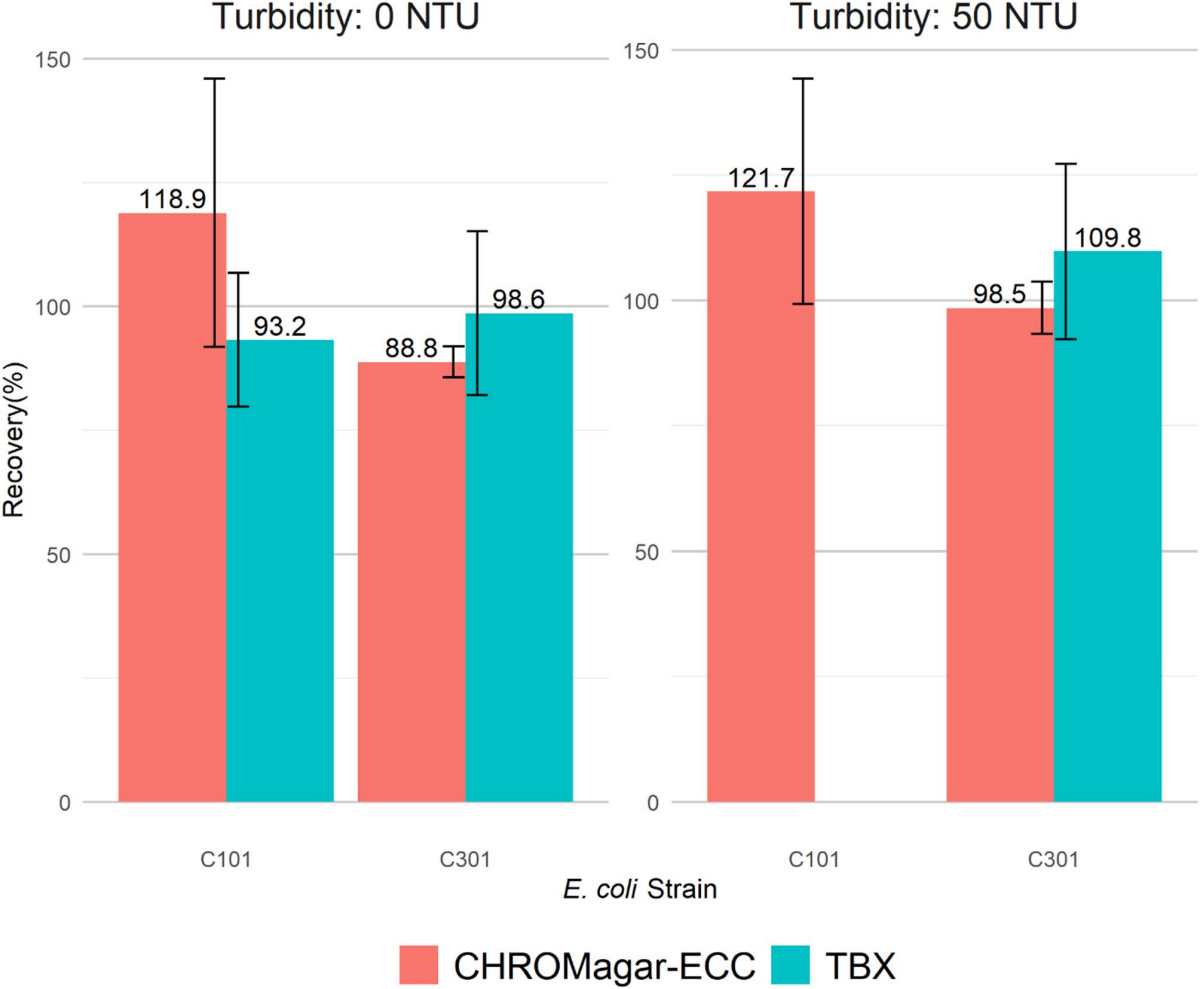
Recovery percentage of *E. coli* ARS C101 (pan-susceptible) and C301 (cefotaxime-resistant) by membrane filtration on selective media. A total of ca. 200 CFU *E. coli* was inoculated in 100 mL Test Agricultural Water (TAW) with turbidity of 0 NTU or 50 NTU. The membrane filter was placed onto two selective media (CHROMagar ECC and TBX) and colony counts were compared with the initial inoculum level to calculate recovery percentage

**Fig. 2:**
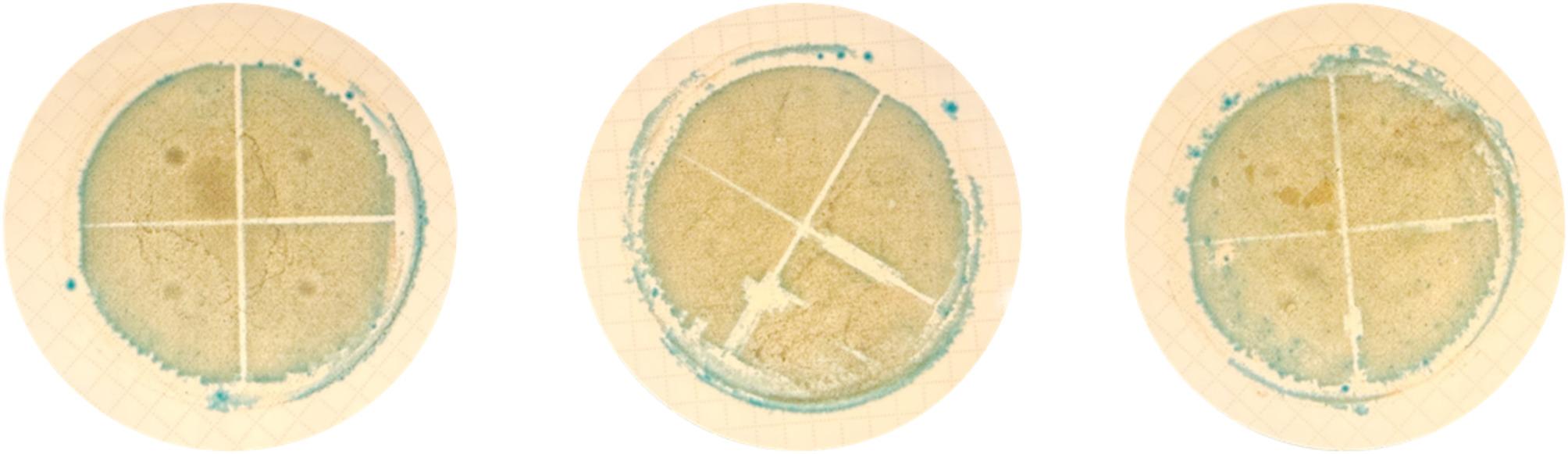
Membrane filters placed onto TBX media to recover *E. coli* ARS C101 in TAW (Test Agricultural Water) at 50 NTU. Growth on membrane filters placed on TBX was not quantifiable because distinct colonies of C101 were not observed

The recovery percentages of *E. coli* ARS C101 by membrane filtration (MF) from TAW with 0 NTU were 93.2 ± 13.5% and 118.9 ± 27.1% on TBX and ECC, respectively (Fig. 1). The recovery percentages of *E. coli* ARS C301 by MF from TAW with 0 NTU turbidity were 98.6 ± 16.5% and 88.8 ± 3.1% on TBX and ECC supplemented with 4 µg/mL cefotaxime, respectively. The recovery percentage of *E. coli* ARS C101 by MF from TAW with 50 NTU turbidity on ECC was 121.7 ± 22.5%. No recovery percentage of C101 by MF from TAW with 50 NTU was observed on TBX due to a “smear effect”, where distinct colonies were not formed (Fig. 2). For all three replicates for C101 recovered on TBX from TAW with 50 NTU, no distinct colonies were observed on the agar plates, perhaps due to the higher turbidity levels in this formulation. Therefore, we were unable to visually detect and quantify the *E. coli* ARS C101 colonies from TAW at 50 NTU. The recovery percentages of *E. coli* ARS C301 by MF from TAW with 50 NTU turbidity were 109.8 ± 17.5% and 98.5 ± 5.2% on TBX and ECC supplemented with 4 µg/mL cefotaxime, respectively.

It is important to evaluate the recovery of pan-susceptible and antibiotic-resistant *E. coli* in a model system since its recovery in natural samples can be influenced by a variety of factors such as geography, season, background microbiota, nutrients, temperature, pH, and UV light exposure [23, 24]. The TAW formula is straightforward, making it a suitable test system that is replicable in laboratory settings. Based on our findings on the recovery of *E. coli* ARS C101 in TAW, we conclude that in agricultural water with higher levels of turbidity, the use of TBX agar with MF may not be reliable for quantification, due to potential interactions between turbidity, the membrane filter, and the TBX media. Large particles and colloids in agricultural water could potentially clog the membrane filter, obstruct filtration and subsequent quantification of bacterial colonies [13]. Previous studies suggested that TBX demonstrated better recovery performance for *E. coli* enumeration through MF compared to other types of selective media based on confirmation tests, with lower false positive and false negative rates for wastewater samples [13, 25–27]. One study that compared the recovery performance of MF with TBX and ECC concluded that both types of media performed equally well for enumeration of *E. coli* in water with high levels of background microorganisms [24]. In their study, the authors considered colonies on TBX the most straightforward to count due to the high contrast in color between chromogenic blue *E. coli* colonies and non-*E. coli* colonies. Others observed that raw or partially treated wastewater with a diverse microbiota is likely to have a higher false positive and false negative rate for both MF methods and the Colilert assay [13]. In general, increasing the selective incubation temperature from 37 °C to 44 °C would increase the selectivity for MF methods from non-sterile sources [28]. Since higher turbidity levels in TAW made it difficult to obtain a countable result on TBX for *E. coli* ARS C101, we concluded that MF method with TBX may not be suitable to quantify certain pan-susceptible *E. coli* isolates when turbidity levels in water are relatively high (50 NTU in this study).

**Fig. 3:**
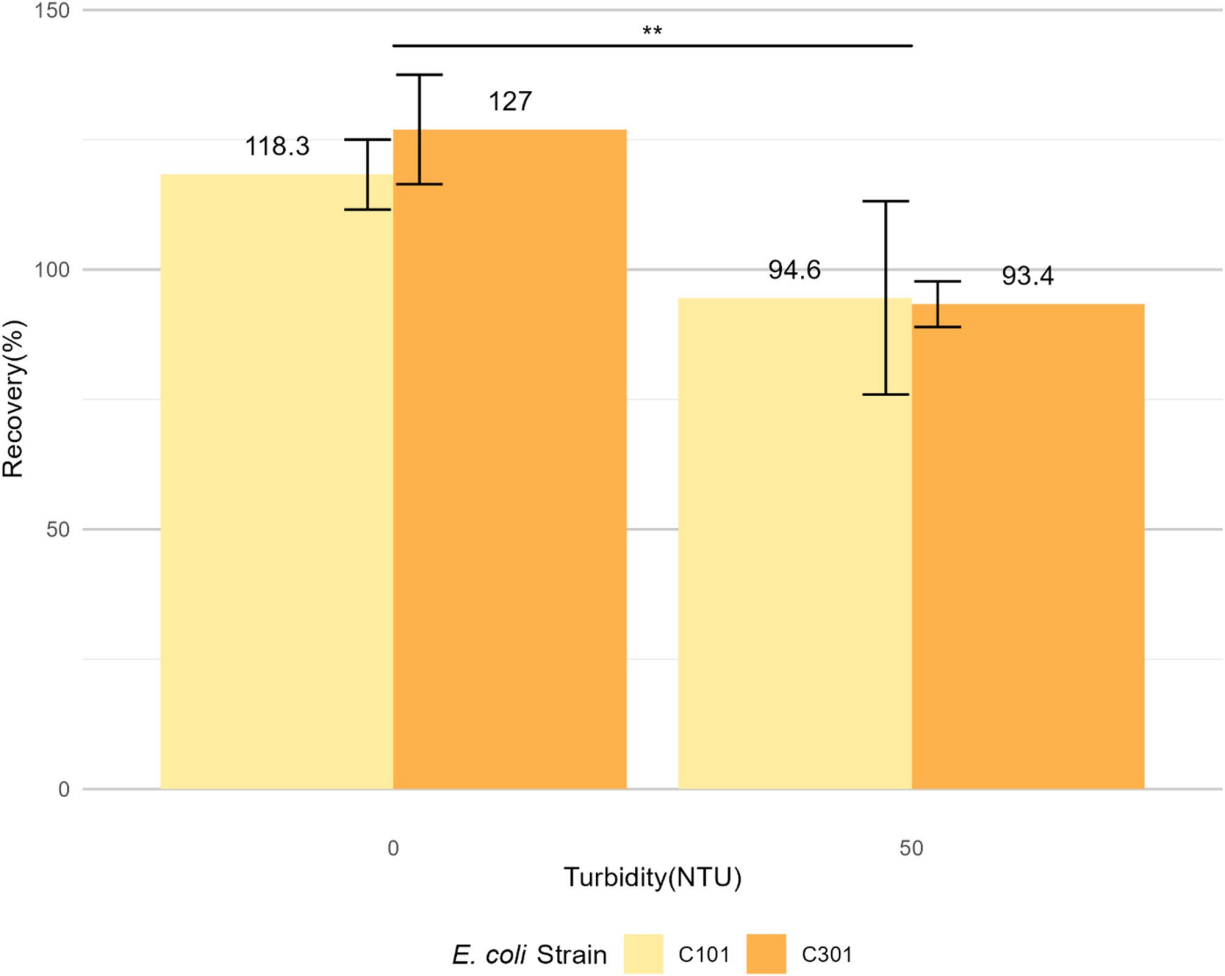
Recovery percentage of *E. coli* ARS C101 and C301 by Quanti-Tray/2000 Colilert assay. A total of ca. 200 CFU *E. coli* was inoculated in 100 mL TAW with turbidity levels of 0 NTU and 50 NTU. The Most Probable Number/100 mL (MPN/100 mL) values of *E. coli* was compared with the initial inoculum level to calculate recovery percentage. Turbidity was a significant factor (*p* = 0.0233, Two-way ANOVA and Tukey HSD test) affecting a higher recovery percentage at 0 NTU compared to at 50 NTU in TAW for the Colilert assay (*p* = 0.0016, Pairwise t-test with Bonferroni correction)

The recovery percentage of *E. coli* ARS C101 by Colilert assay from TAW with 0 NTU turbidity was 118.3 ± 6.7%, while the recovery from TAW with 50 NTU turbidity was 94.6 ± 18.6% (Fig. 3). The recovery percentage of *E. coli* ARS C301 by Colilert assay from TAW with 0 NTU turbidity was 127.0 ± 10.5%, while the recovery from TAW with 50 NTU turbidity was 93.4 ± 4.4%.

**Fig. 4:**
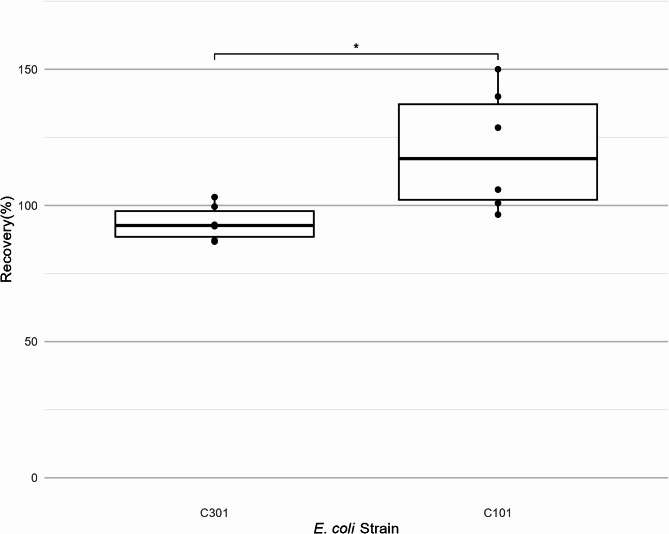
Strain type is a significant factor for recovery by membrane filtration with CHROMagar ECC. *P*-value is 0.0233 based on two-way ANOVA and Tukey HSD test. Pairwise t-test showed that the difference between recovery percentage of *E. coli* ARS C101 and C301 was significant (*p* = 0.03, *p*-value with Bonferroni correction)

For membrane filtration, strain-based differences in recovery percentages were observed, but turbidity levels nor recovery medium affected levels of recovery percentages. The recovery percentage of C101 was significantly greater (*p* = 0.0233) than that of C301 on ECC, based on two-way ANOVA and Tukey HSD test (Fig. 4). Similar effects from strain variations for MF method were reported in previous studies [15, 29].

For the Colilert assay, turbidity was a significant factor which affected recovery percentages of both ARS C101 and C301, based on two-way ANOVA and Tukey HSD test (Fig. 3). The higher turbidity level of 50 NTU significantly (*p* = 0.0016) decreased the recovery percentages of *E. coli* ARS C101 and C301 compared to recovery percentages at lower turbidity level (0 NTU). Previous studies considered Colilert and MF methods equivalent to detect *E. coli* considering sensitivity and specificity, especially in environmental matrices with potential growth inhibitors [13, 15, 25].

To combat the global health threats of antimicrobial resistance, monitoring levels of pan-susceptible and ESBL-producing *E. coli* is crucial. Colilert is an accessible method that requires little training and minimal lab equipment to perform, particularly suitable for developing countries and remote areas, bridging the gap on the AMR surveillance data in different countries [15]. Colilert was considered a superior enumeration method for turbid samples since the fluorescence of presumptive *E. coli* was thought to overcome potential interference of high levels of turbidity. Similarly, higher levels of turbidity in water samples may clog filters and inhibit colony formation by MF methods [15]. In our study, we observed that higher turbidity levels decreased the recovery percentage of *E. coli* in test agricultural water when using the Colilert assay.

## Limitations

Only two *E. coli* strains and two turbidity levels were evaluated. Test agricultural water with turbidity levels exceeding 50 NTU were not tested in this study. The specific threshold of turbidity level between 0 and 50 NTU agricultural water that may interfere with enumeration of certain *E. coli* strains by MF with TBX was not determined. Future studies should explore the recovery percentage of different *E. coli* strains under different levels of turbidity in both environmental and test agricultural water using MF and Colilert assays.

## Conclusion

Our study demonstrated that membrane filtration with ECC and Colilert assay provided quantifiable results for recovery of pan-susceptible and cefotaxime-resistant *E. coli* from test agricultural water at different turbidity levels. A higher turbidity level in test agricultural water interfered with the quantification of pan-susceptible *E. coli* strains when using membrane filtration with TBX media and decreased the recovery percentage with Colilert assay.

## Supplementary Information

Below is the link to the electronic supplementary material.

Supplementary Material 1

## Data Availability

No datasets were generated or analysed during the current study.

## Abbreviations

AMR: Antimicrobial resistance
ECC: CHROMagar ECC
EPA: Environmental protection agency
ESBL: Extended-spectrum beta-lactamase
MF: Membrane filtration
MPN: Most probable number
TAW: Test agricultural water
TBX: Tryptone bile X-glucuronide
TDS: Total dissolved solids
TOC: Total organic carbon
TSA: Tryptic soy agar
TSB: Tryptic soy broth
TSBC: Tryptic soy broth with 4 µg/mL cefotaxime
