# Supplementary material for: Evaluating the recovery of pan-susceptible and antibiotic-resistant Escherichia coli in synthetic test agricultural water using membrane filtration and colilert methods
Source: BMC Res Notes. 2025 Aug 20;18:365. doi: 10.1186/s13104-025-07423-9 (PMC12366208; doi:10.1186/s13104-025-07423-9)
Supplement: Supplementary file 1 — Supplementary Material 1 [file 13104_2025_7423_MOESM1_ESM.docx]

**Table S1. Synthetic Test Agricultural Water (TAW) formulation (adapted from EPA protocol Reg. No. 94151PA7).**

| Component | Quantity | Target Parameter | Target Value |
| --- | --- | --- | --- |
| Sterile deionized water | 1000 mL | Total chlorine | <0.02 mg/L |
| PTI Arizona Test Dust  (Nominal 0-70 micron) | 0 g (0 NTU)  or  0.35 g (50 NTU) | Turbidity | 0  or  50 NTU |
| Humic Acid | 10 mg | Total organic carbon | >10 mg/L |
| Sea Salts | 1.6 g | Total dissolved solids | 1350-1650 mg/L |
| 1 M HCl and/or 1 M NaOH | As needed | pH | 6.5 |
